# Open Surgical Decompression for Large Multiloculated Spinoglenoid Notch Ganglion Cyst With Suprascapular Nerve Neuropathy

**DOI:** 10.7759/cureus.13300

**Published:** 2021-02-12

**Authors:** Terrence Jose Jerome, Vanathi Sabtharishi, Thirumagal SK

**Affiliations:** 1 Orthopaedics, Hand and Reconstructive Microsurgery, Olympia Hospital and Research Centre, Trichy, IND; 2 Microbiology, KAP Viswanatham Medical College and Hospital, Trichy, IND; 3 Trauma, Olympia Hospital and Research Centre, Trichy, IND

**Keywords:** open surgical decompression, large multiloculated ganglion cyst, spinoglenoid cyst, suprascapular neuropathy

## Abstract

Suprascapular nerve entrapment at the spinoglenoid notch causes infraspinatus weakness and wasting. Patients present with shoulder pain and weakness. The spinoglenoid notch cyst is the reason for suprascapular nerve compression. Magnetic resonance imaging (MRI) confirms the diagnosis of spinoglenoid cyst and its nerve compression. Also, MRI rules out other differential diagnosis causing shoulder pain and weakness. One of the treatment modalities for small and asymptomatic cyst is conservative, which has produced acceptable results and functional outcome. Open or arthroscopic aspiration or decompression is indicated for patients with single small cysts where conservative treatment failed, and cyst associated with suprascapular nerve compression. We report a 32-year-old dancer with a large multiloculated multiple spinoglenoid cysts compressing the suprascapular nerve causing infraspinatus wasting and shoulder dysfunction. We performed an open surgical decompression of the suprascapular nerve and excised multiple ganglions. The patient improved significantly and regained his shoulder function and muscle wasting at two-year follow-up.

## Introduction

The common etiology for shoulder pain and weakness in adults and old alike are rotator cuff tears, capsulolabral tears, tendinitis, adhesive capsulitis, acromioclavicular joint osteoarthritis and cervical diseases such as disc prolapse and spondylosis [1]. Suprascapular nerve entrapment has recently attracted the attention of orthopaedics and hand surgeons where patients present with posterior shoulder pain and weakness. The suprascapular nerve is commonly entrapped at the suprascapular notch to cause supraspinatus and infraspinatus weakness and wasting [2]. The nerve can also be trapped at the spinoglenoid notch, where the infraspinatus alone is involved [3].

Ganglion cysts, trauma, shoulder dislocations, proximal humerus fractures, scapular fractures, iatrogenic injury, traction injuries or stretching of spinoglenoid ligaments in extreme cross-body adduction and internal rotation of the shoulder joint can cause suprascapular nerve neuropathy at the spinoglenoid notch [1,4-7].

Several authors have reported solitary spinoglenoid cyst as a common reason for suprascapular nerve entrapment. The patients have also associated posterior capsulolabral tears, which contribute to cyst formation through a valve-like mechanism [7-10]. Conservative treatment such as rest, analgesics, and minimally invasive cyst aspiration followed with physiotherapy for three months have shown good functional improvement [1,7,11]. Few case reports documented that open or arthroscopic cyst decompression and labral repairs produced excellent shoulder functions [7-11].

We report a unique case of multiple large ganglion cysts in the spinoglenoid notch causing suprascapular neuropathy in a dancer. Open surgical decompression produced excellent pain relief and regained his full shoulder functions. 

## Case presentation

**Figure 1:**
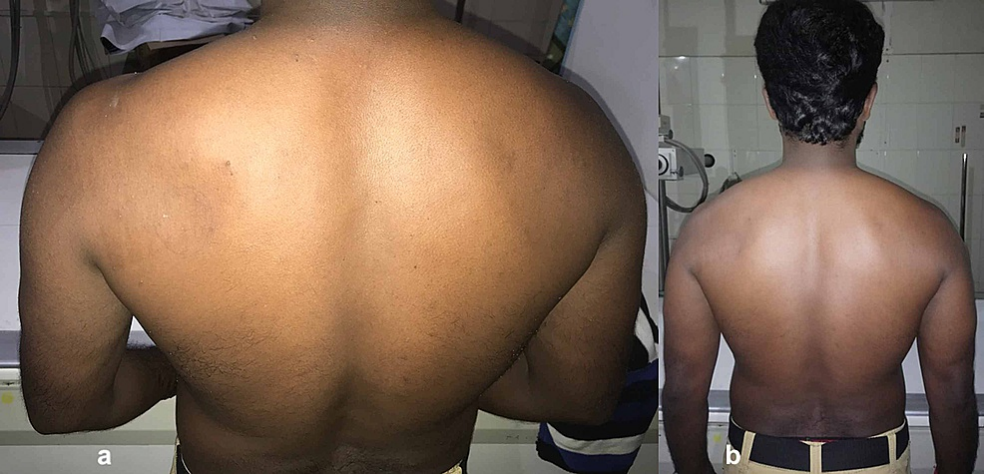
Clinical picture of left shoulder showing the wasting seen in infraspinatus fossa and reduced muscle bulk.

A 32-year-old dancer presented with the complaint of left posterior shoulder pain and weakness for eight months. He also noted the difficulty in performing various dance steps (glide, capoeira and salsa), particularly initiating any overhead activities and pain during sleeping on the left shoulder. He had no trauma or shoulder injury. On physical examination, we found infraspinatus muscle wasting and tenderness over the spinoglenoid area (Figure 1).

**Figure 2:**
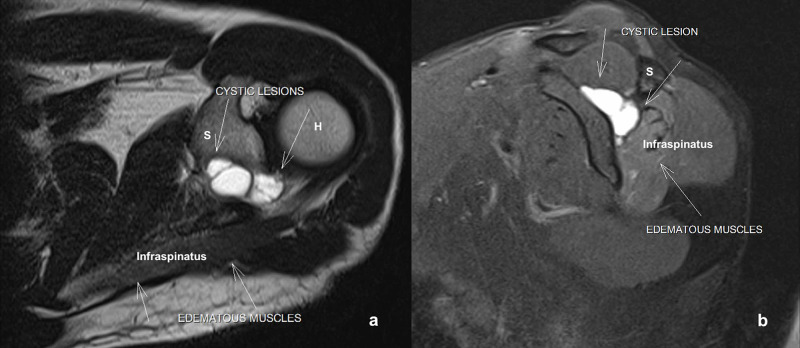
Magnetic resonance imaging. (a) T2 axial section showing multiple multiloculated spinoglenoid cyst (white arrow) in the left shoulder; H - humerus head; S - spine of scapula. (b) Sagittal section showing spinoglenoid cyst with the scapular spine (S) of the left shoulder and denervation of the infraspinatus muscle.

We also noted the absence of shoulder external rotation and weak abduction. Neck movements, elbow and hand, were clinically normal. Radiographs of the left shoulder were inconclusive. MRI showed multiple loculated ganglion cysts (3x2x3 cm largest size) in the spinoglenoid notch compressing the suprascapular nerve (Figure 2).

The infraspinatus muscles were edematous and atrophic with minimal fatty infiltration consistent with denervation changes. The shoulder joint, rotator cuff, capsule, glenoid labrum and acromioclavicular joints were normal. Electromyography and nerve conduction velocity (EMG/NCV) showed the motor loss in infraspinatus, denervation potentials, prolonged motor latency, delay in conduction time and reduction in the velocity.

**Figure 3:**
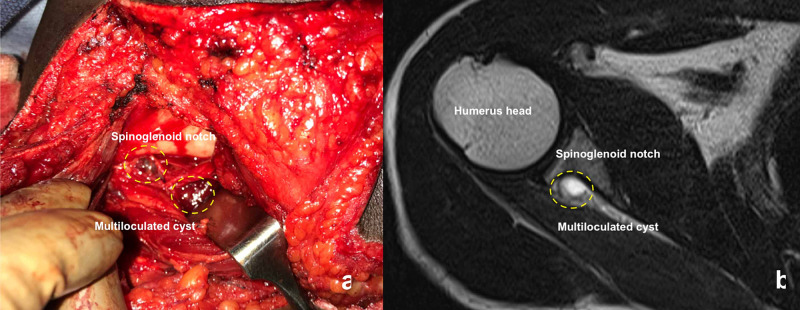
(a) Intraoperative pictures showing the spinoglenoid notch and multiple cysts (yellow dotted circle). (b) MRI images correlating the intraoperative pictures showing the spinoglenoid notch, cysts (yellow dotted circle) and humeral head.

Considering the large size and multiple loculated ganglion, we preferred open decompression of the cyst using the posterior approach with the patient in the prone position. We retracted the infraspinatus muscle and reached the spinoglenoid notch. We noted multiple large bluish-black discolored ganglions were compressing the suprascapular nerve (Figure 3).

**Figure 4:**
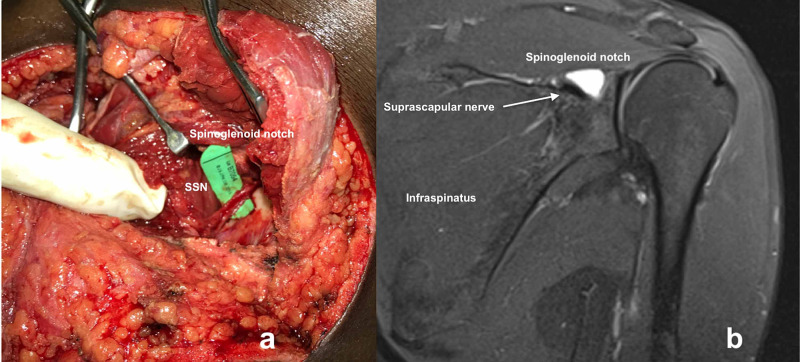
(a) Intraoperative pictures showing the spinoglenoid notch and decompressed suprascapular nerve (SSN). (b) MRI images correlating the intraoperative pictures showing the spinoglenoid notch, cysts and decompressed suprascapular nerve. The nerve runs inferolateral in the spinoglenoid notch and curves around the lateral margin of the scapular spine to enter the infraspinatus fossa.

We gently isolated the cysts from the nerve and excised all the ganglion cysts. Surgeons must take care to prevent iatrogenic suprascapular nerve injury. The nerve runs inferolateral in the spinoglenoid notch and curves around the lateral margin of the scapular spine to enter the infraspinatus fossa (Figure 4).

**Figure 5:**
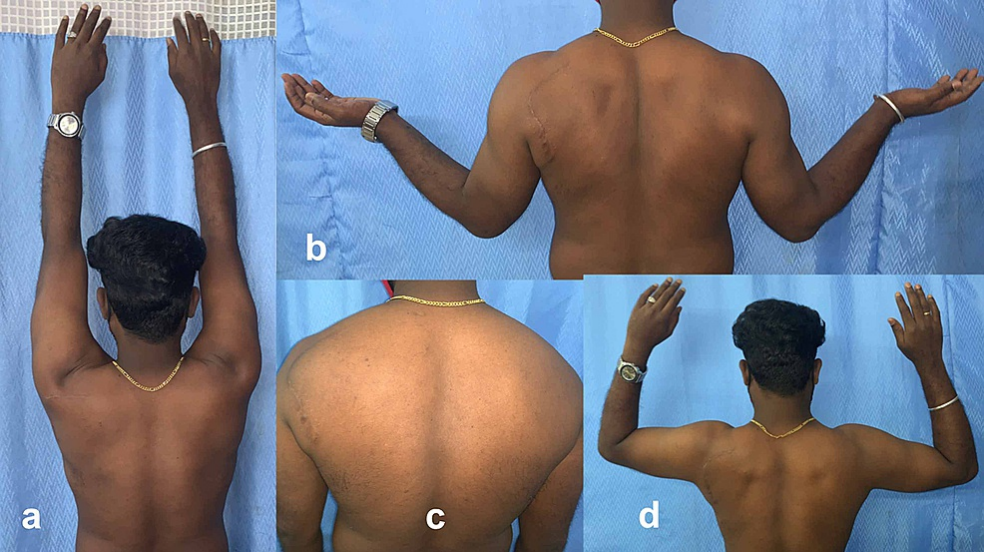
Follow-up clinical pictures (a-d) showing good shoulder functions (abduction, external rotation) and regained infraspinatus muscle mass.

Usually, the ganglion is seen superior to the nerve, and most surgeons perform decompression and prefer not to visualize the suprascapular nerve. Since we had large and multiple cysts, we identified and isolated the suprascapular nerve along with cyst excision. Postoperatively, we immobilized the patient in a broad arm sling for two weeks and then removed the sutures. We put him on regular physiotherapy and did not restrict many movements. The patients became asymptomatic and slowly regained muscle wasting. He had excellent shoulder function (abduction, external rotation) with usual infraspinatus muscle bulk at two-year follow-up (Figure 5).

EMG showed complete axonal reinnervation and normal action potential for infraspinatus muscle.

## Discussion

Various authors have described the clinical presentation, diagnosis and treatment modalities for solitary spinoglenoid cyst with or without labral tears. Shoulder pain (100%), and weakness (67%) are the presenting complaints, and infraspinatus atrophy (40%) and posterior shoulder tenderness (48%) are classical clinical findings [11]. MRI is the diagnostic modality for spinoglenoid cyst and associated rotator cuff and labral defects [1,2,9-11]. Also, fatty infiltration and wasting are visualized in an MRI. EMG/NCV assists the clinical diagnosis of infraspinatus atrophy and differentiates from suprascapular notch entrapment and cervical disc prolapse.

Small-sized cysts with minimal symptoms, no shoulder pathologies and absent muscle wasting are managed with rest, NSAIDs (non-steroidal anti-inflammatory drugs) and physiotherapy. These patients regain back to useful shoulder function in three months. Posterior labral tear of the glenoid with unidirectional shoulder instability, decreased range of motion with supraspinatus and infraspinatus muscle weakness are treated with arthroscopic labrum fixation and cyst decompression [10]. These patients achieve good to excellent shoulder stability, movements and regain muscle mass [8-10]. Arthroscopic labral repair closes the valve-like mechanism, which caused the leak of synovial fluid from the shoulder joint after labral tears into extra-articular space (spinoglenoid cyst). This is an indirect method of decompressing the cyst with a risk of spinoglenoid cyst recurrence [8-12].

In more than 53% of cases with spinoglenoid cyst, MRI shoulder may not detect. Arthroscopic or ultrasound-guided drainage may be useful in such conditions [12]. Recurrence up to 48 % is inevitable within two years [1]. Many authors have believed that not draining the spinoglenoid cyst during the arthroscopic labral repair allows the cysts to self-absorb over time [9,10]. Intra-articular arthroscopic decompression by blunt dissection and suction is not always possible in large multiloculated cysts. Many authors prefer open incision than arthroscopic repair in cases with nerve compression and weakness [1,11,13,14].

Open surgical decompression allows direct visualization of the cyst, complete cyst excision, and prevents recurrence in normal shoulder labrum cases. In patients with symptoms and small cyst, aspiration may decompress the nerve entrapment [1,11]. Unfortunately, cyst recurrence may occur [15]. The suprascapular nerve lies close (18 to 21 mm) to the glenohumeral joint at the spinoglenoid notch level [16]. Surgeons should exercise care in preventing iatrogenic injury to the nerve while open decompression. A combination of arthroscopic debridement and open cyst excision is also possible. This has been shown to have comparable results with low recurrence rates [11,17].

Our patient had significant shoulder pain during his dance movements and weakness. On physical examination, he had posterior shoulder tenderness, external rotation weakness, and infraspinatus atrophy. MRI confirmed the diagnosis and defined the margins of a large multiloculated multiple ganglion cyst. Also, the posterior labrum was normal. The treatment's goal was to resolve the patient's symptoms, which was accomplished by open decompression of the cyst. Our patient was completely satisfied. He regained normal shoulder function and reinnervation of infraspinatus muscle mass at two years.

## Conclusions

Spinoglenoid notch cyst is rare and sometimes associated with rotator cuff conditions and posterior labral tears. Large multiloculated spinoglenoid ganglion cyst with normal rotator cuff and glenoid labrum is not reported. Open decompression of multiple large cysts produces excellent shoulder function, allows the atrophied muscle to regenerate, and reduces recurrence risk. Suprascapular neuropathy secondary to large multiloculated cysts can be safely treated with open surgical decompression with potentially good results.
